# Correction: PPanGGOLiN: Depicting microbial diversity via a partitioned pangenome graph

**DOI:** 10.1371/journal.pcbi.1009687

**Published:** 2021-12-10

**Authors:** Guillaume Gautreau, Adelme Bazin, Mathieu Gachet, Rémi Planel, Laura Burlot, Mathieu Dubois, Amandine Perrin, Claudine Médigue, Alexandra Calteau, Stéphane Cruveiller, Catherine Matias, Christophe Ambroise, Eduardo P. C. Rocha, David Vallenet

There is an error in S3 File, “List of GenBank assembly accessions for the 439 studied species.” The list of assembly accessions was truncated. Please see the complete file below:

## Supporting information

S3 File List of GenBank assembly accessions for the 439 studied species. This is a TSV file where each line corresponds to all the GenBank assembly accession used in this study for each ‘species id’ in the NCBI taxonomy.
(TSV)

## References

[1] Gautreau G, Bazin A, Gachet M, Planel R, Burlot L, Dubois M, et al. (2020) PPanGGOLiN: Depicting microbial diversity via a partitioned pangenome graph. PLoS Comput Biol 16(3): e1007732. 10.1371/journal.pcbi.1007732 32191703 PMC7108747

