# Supplementary figures and images for: PPanGGOLiN: Depicting microbial diversity via a partitioned pangenome graph
Source: PLoS Comput Biol. 2020 Mar 19;16(3):e1007732. doi: 10.1371/journal.pcbi.1007732 (PMC7108747; doi:10.1371/journal.pcbi.1007732)

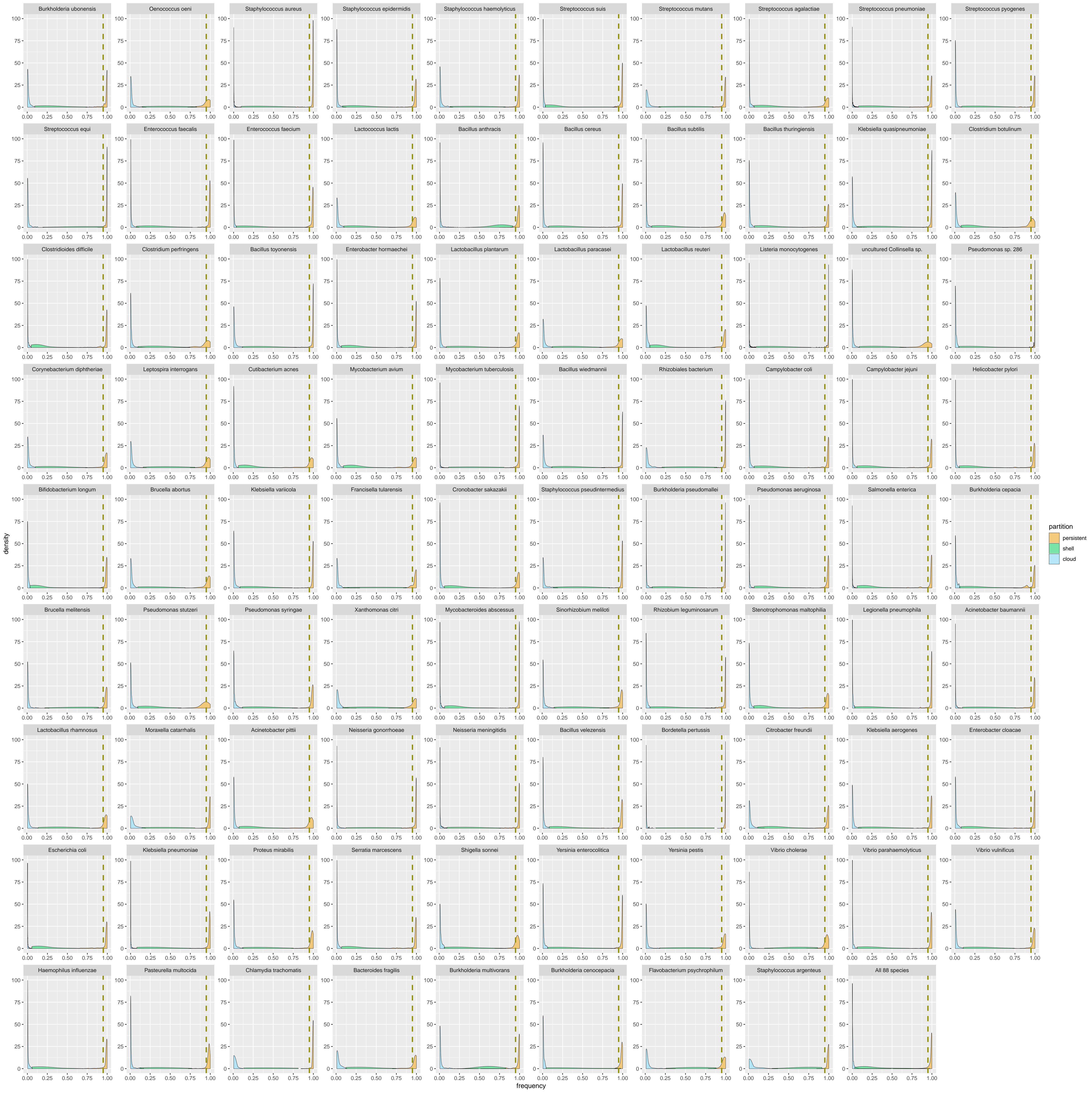

Supplement: S1 Fig — Results for the 88 most abundant species in GenBank are represented in addition with a global distribution of the gene family frequencies from all the species. Density values of the cloud genome above 100 (y-axis) were trimmed for visualization purpose. The dashed yellow vertical bars indicate the threshold of frequency (⩾95%) used to delimit the soft core genome. (PDF) [file pcbi.1007732.s001.pdf]

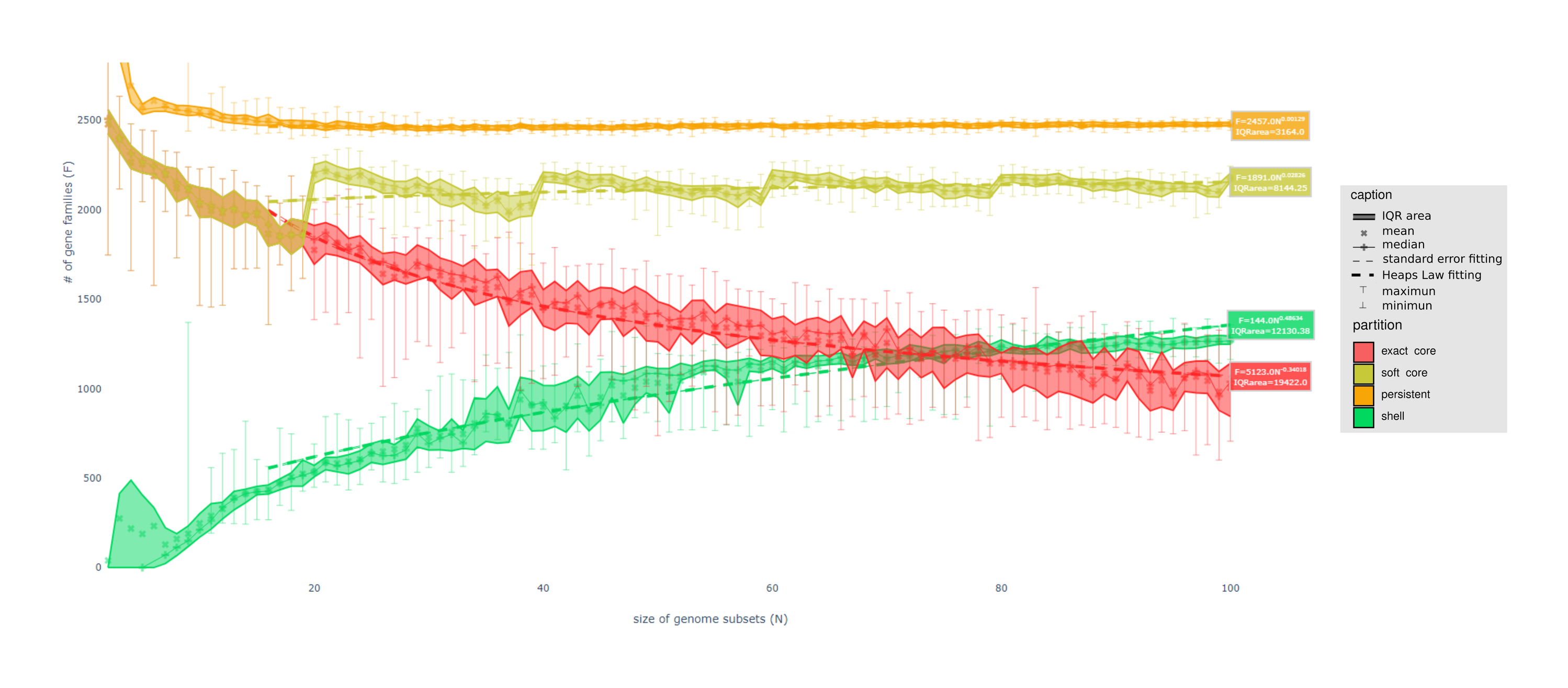

Supplement: S2 Fig — The rarefaction curves represent the evolution of the partition sizes as a function of an increasing number of genomes in random subsets of genomes. Plain lines connect the medians while colored areas represent the interquartile ranges. A regression curve (bold dashed line) is drawn fitting all the points of each partition by the Heaps’ law (F = κNγ). The total area of the interquartile ranges (IQR) is indicated for each partition. (TIF) [file pcbi.1007732.s002.tif]

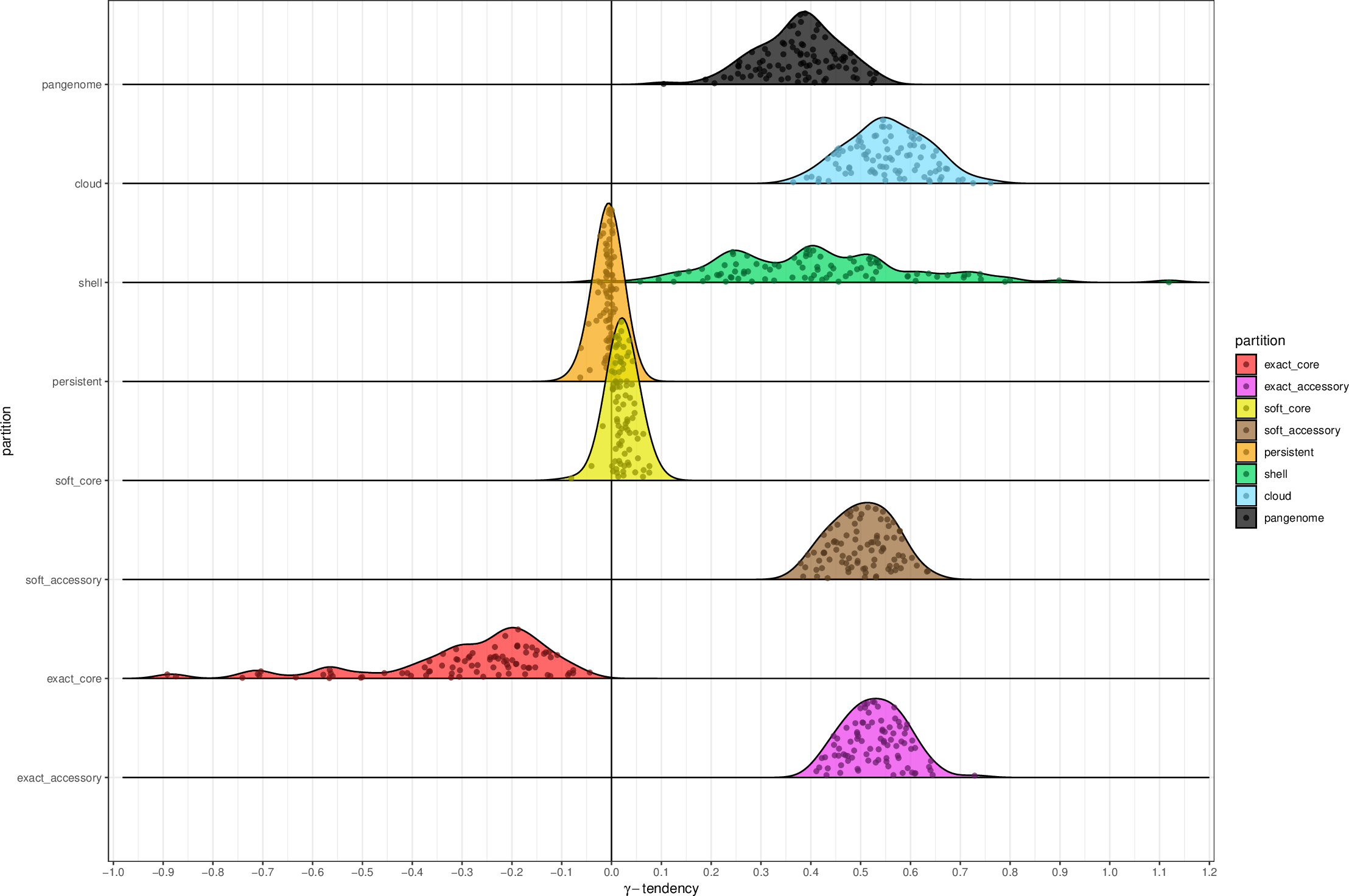

Supplement: S3 Fig — These γ-tendencies were obtained by fitting a Heaps’ law on rarefaction curves between subset sizes of 15 to 100 genomes in the 88 most abundant species in GenBank. The exact core median and exact accessory are not shown. (TIF) [file pcbi.1007732.s003.tif]

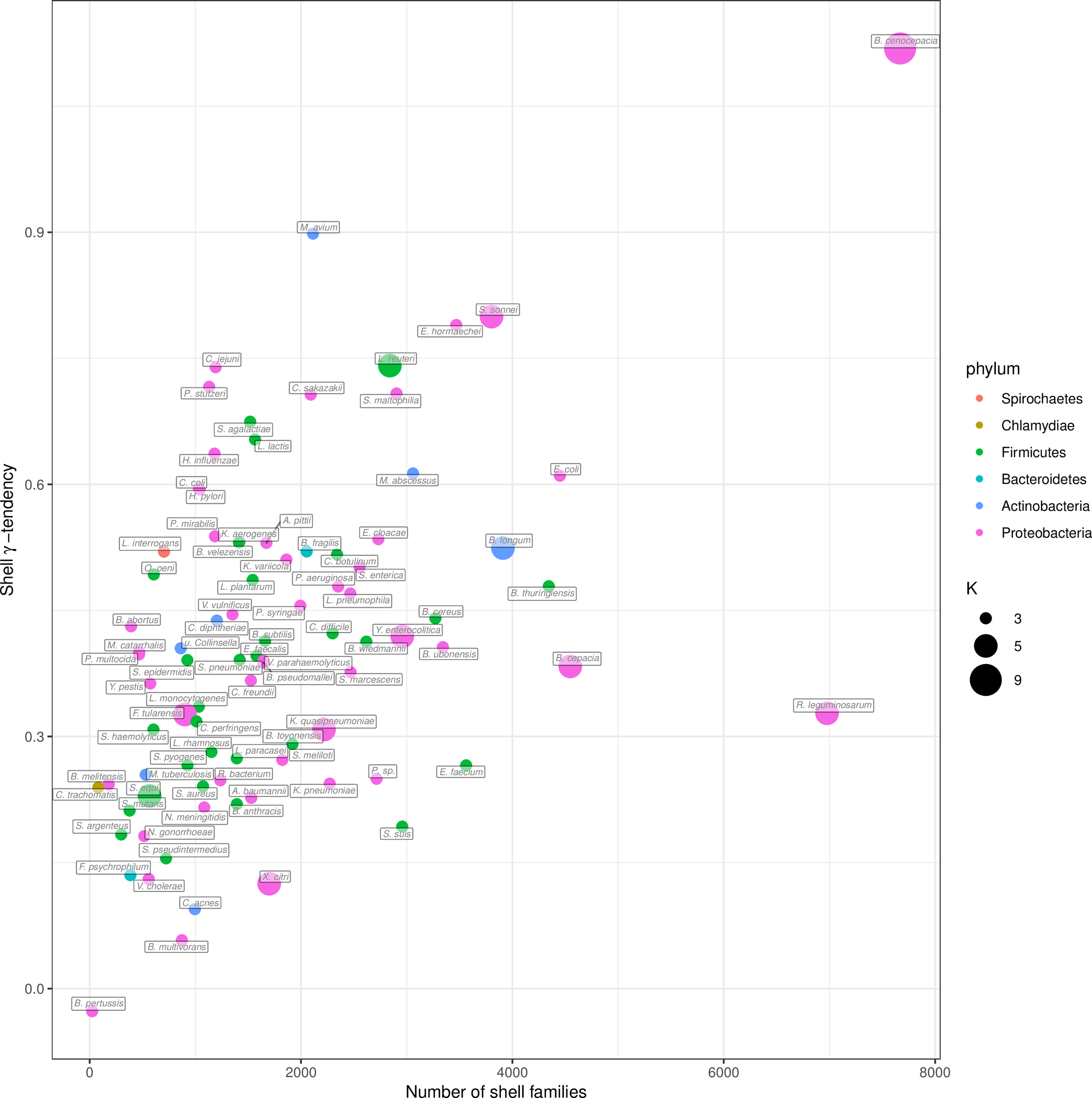

Supplement: S4 Fig — Results for the 88 most abundant species in GenBank are represented. The points are colored by phylum and their size corresponds to the number of partitions (K) used. (TIF) [file pcbi.1007732.s004.tif]

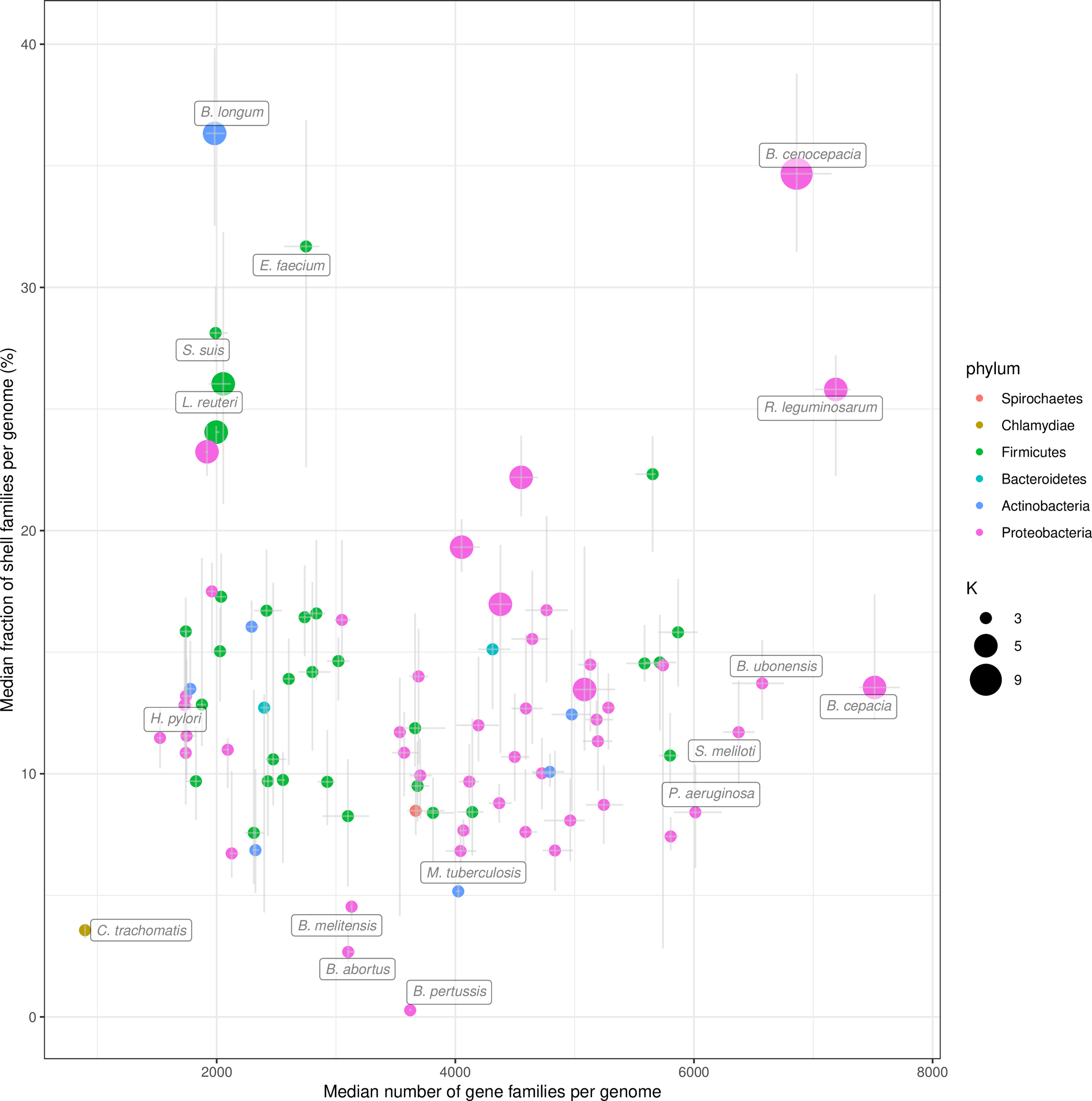

Supplement: S5 Fig — Results for the 88 most abundant species in GenBank are represented. The points are colored by phylum and their size corresponds to the number of partitions (K) used. (TIF) [file pcbi.1007732.s005.tif]

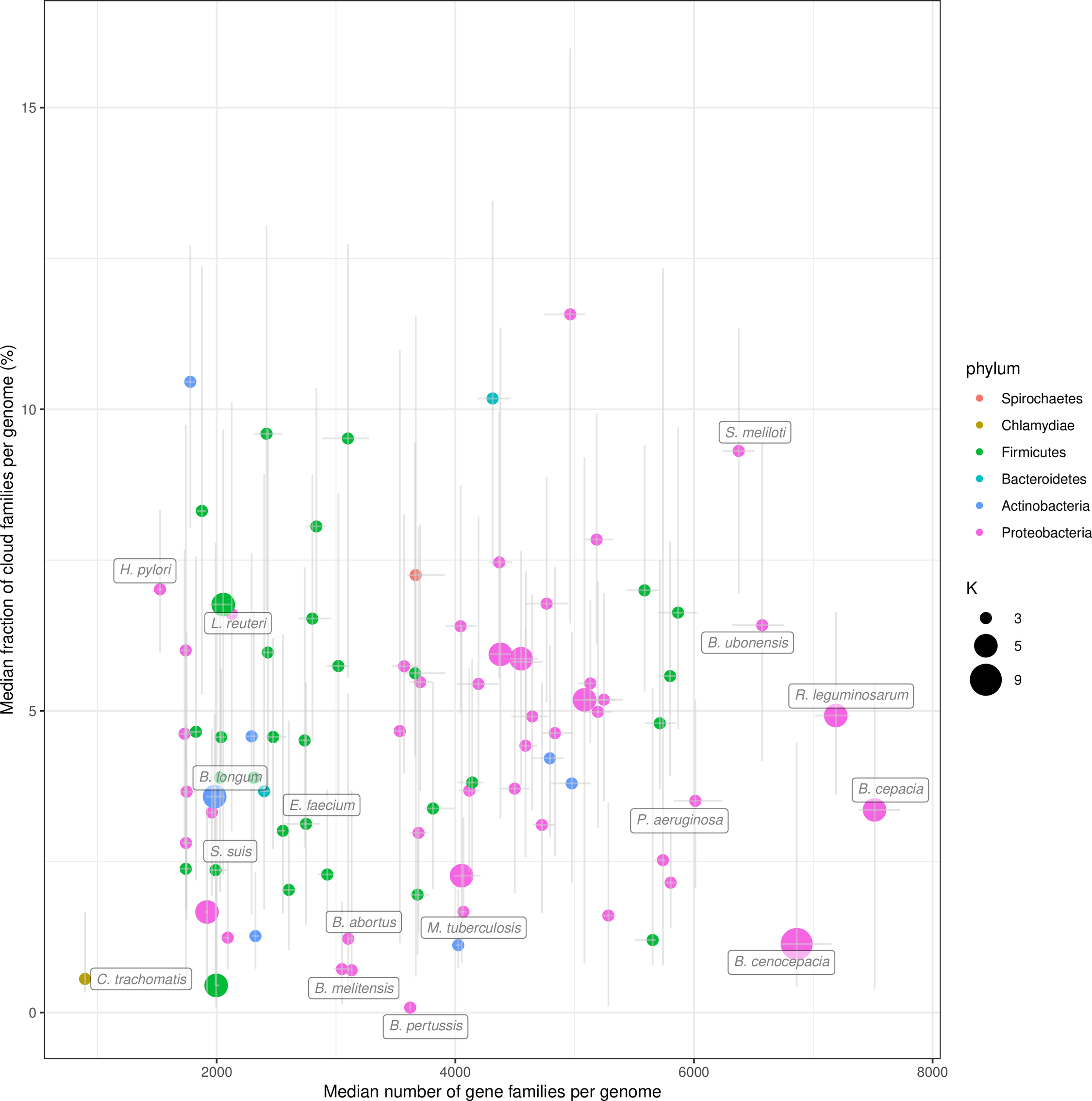

Supplement: S6 Fig — Results for the 88 most abundant species in GenBank are represented. The points are colored by phylum and their size corresponds to the number of partitions (K) used. (TIF) [file pcbi.1007732.s006.tif]

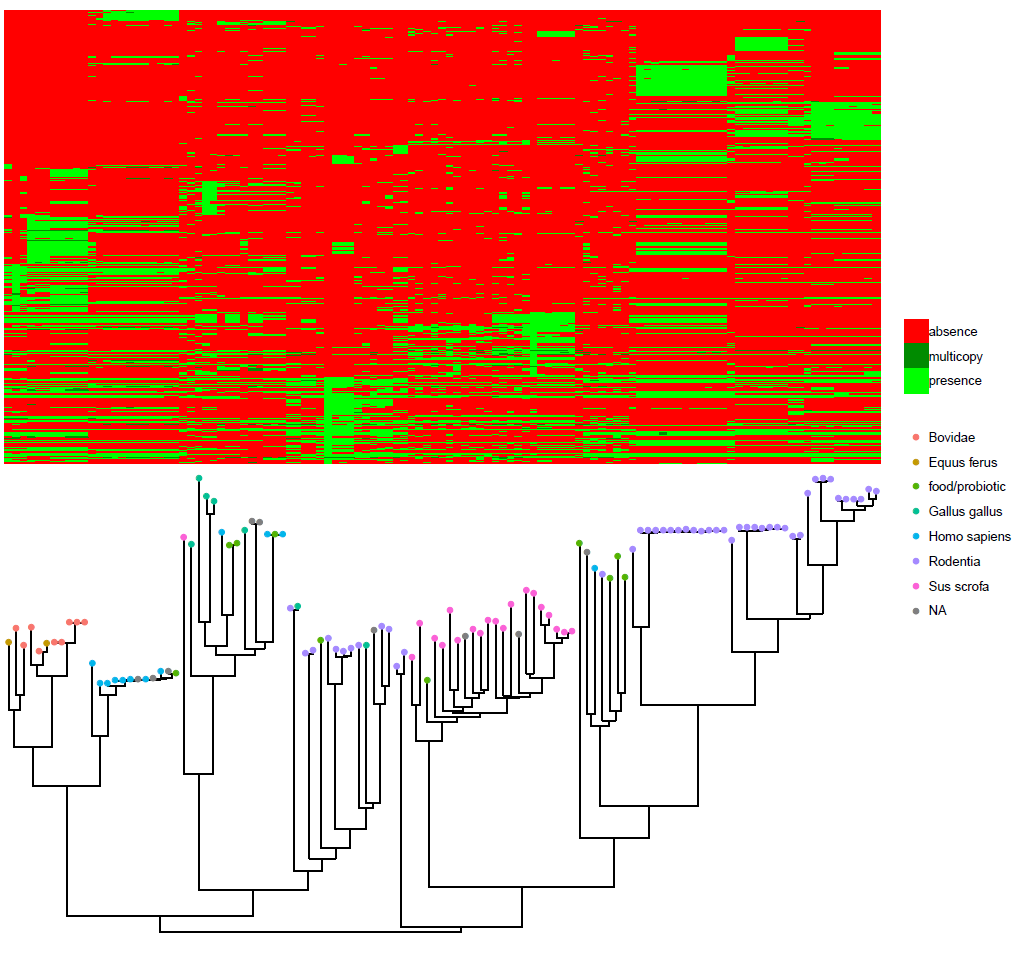

Supplement: S7 Fig — The leaves of the tree are colored by host or origin. This information was obtained from the metadata in GenBank files (host and isolation source qualifiers). (TIF) [file pcbi.1007732.s007.tif]

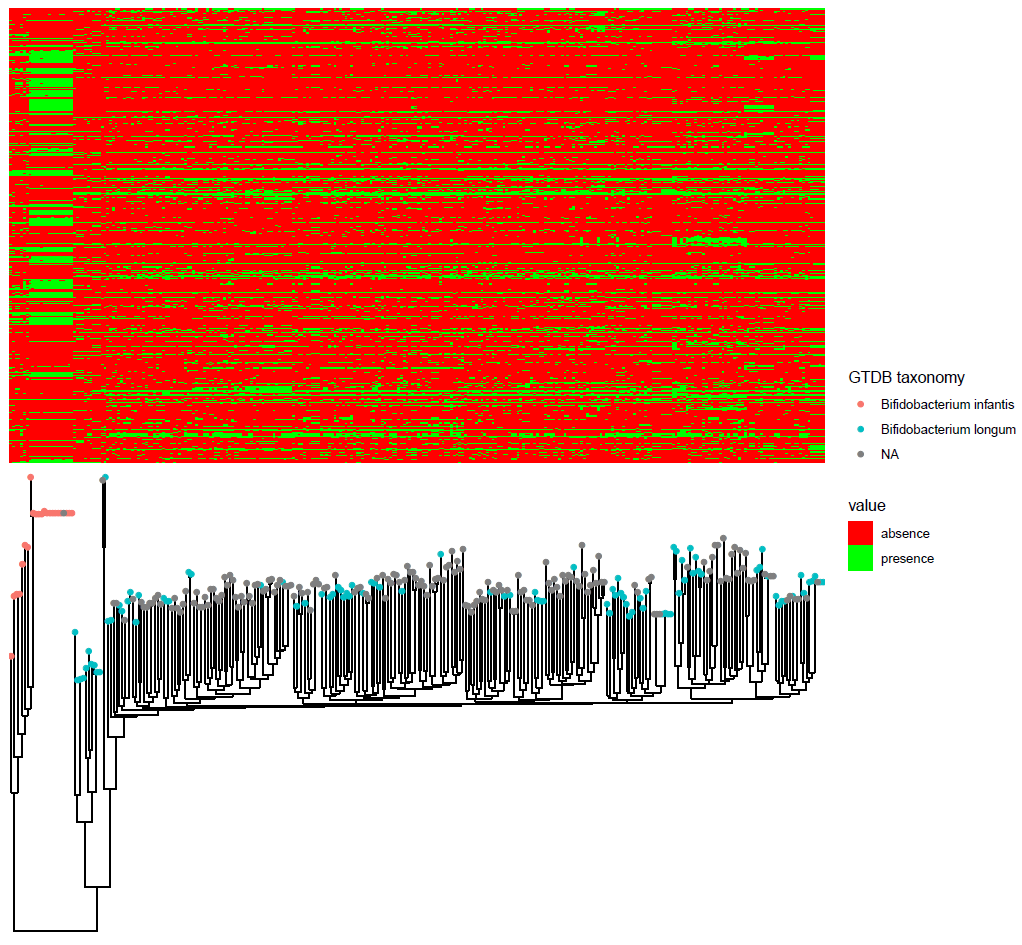

Supplement: S8 Fig — The leaves of the tree are colored by species clusters defined by the GTDB database (release R04-RS89), namely (B. infantis or B. longum). “NA” values correspond to genomes not available in GTDB. (TIF) [file pcbi.1007732.s008.tif]

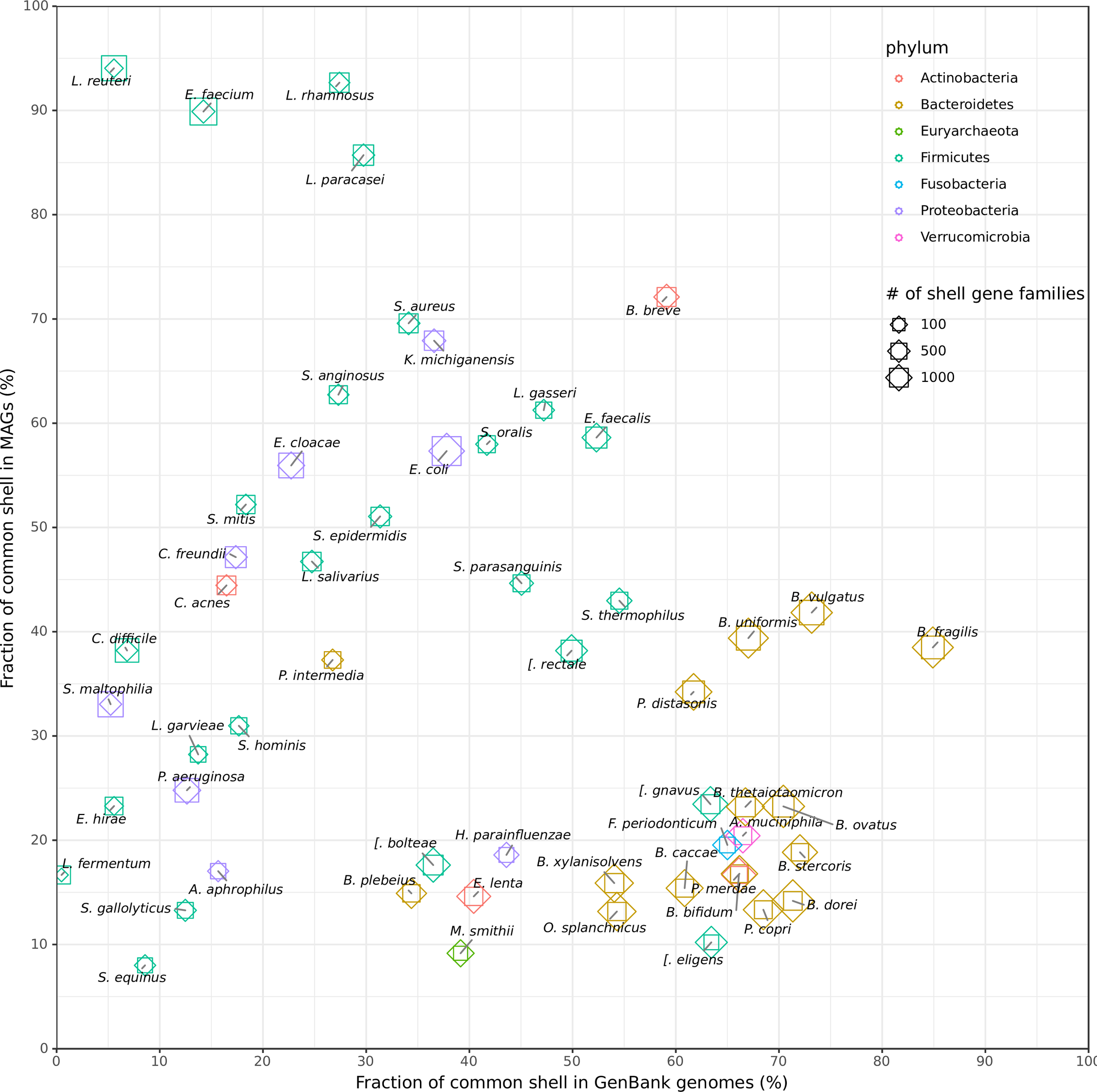

Supplement: S9 Fig — The x-axis represents the percentage of common shell of the GenBank shell while the y-axis corresponds to the percentage of common shell of the MAGs shell. Diamonds and squares represent MAGs and GenBank genomes, respectively. They are colored by phylum and their size indicates the number of genomes. (TIF) [file pcbi.1007732.s009.tif]
