# Supplementary material for: PPanGGOLiN: Depicting microbial diversity via a partitioned pangenome graph
Source: PLoS Comput Biol. 2020 Mar 19;16(3):e1007732. doi: 10.1371/journal.pcbi.1007732 (PMC7108747; doi:10.1371/journal.pcbi.1007732)
Supplement: Attachment — Submitted filename: Authorss responses to reviewers.docx [file pcbi.1007732.s013.docx]

**Authors Responses to reviewers**

First of all, we wish a happy new year to the editors and the reviewers.

**Reviewer #1:** "PPanGGOLiN: depicting microbial diversity via a partitioned pangenome graph", authored by G. Gautreau et. al. presents PPanGGoLiN; a graph-based method for the classification of gene families into persistent, cloud and one or several shell partitions,

that can be used for prokaryotic genomes. The authors make a strong case on the relevance of the persistent genome instead of the more frequently used soft core genome. Moreover, they point out the value of the shell genome in the study of evolutionary dynamics. They also

demonstrate the robustness of their method by applying it to metagenome-assembled genomes and comparing their results to the soft core metric.

The article is well written and the results are sufficiently supported by the text and figures. There are a few points, however, where I would have liked some more clarifications and which are described in detail below.

**Answer 1.0 : We thank the reviewer #1 for his positive comments.**

**Action 1.0 : None**

1-- For the illustration of the partitioned pangenome graph you analysed the A. baumannii pangenome (line 107). There you report the sizes of persistent, shell and cloud values derived from your method. How do you evaluate these numbers? Are they expected? How do they compare to existing literature (for example Chan 2015 which you mention in the introduction, or Hassan 2016)?

**Answer 1.1 : We agree with the reviewer #1 that it would be of interest to compare our predictions with the ones of Chan et al. 2015.**

**According to Chan et al., the core genome of *A. baumannii* using 249 genomes is estimated between 2 833 and 3 126 gene families depending on the threshold of presence used (95% -> 75% of presence). According to the PPanGGOLiN method, we found a persistent genome containing 3 084 gene families using 3 117 genomes. PPanGGOLiN results are thus similar to the ones of Chan et al. if we compare the persistent with the soft-core. For Hassan et al. 2016 paper, only the strict core on 30 complete genomes is provided (2 445 gene families) and thus it cannot be compared to our results because the strict core is very dependent on the number of genomes that are considered even using complete genomes.**

**Action 1.1 : We added sentences about Chan et al. results at the beginning of the “Illustration of a Partitioned Pangenome Graph depicting the Acinetobacter baumannii species” subsection. We also corrected a small error in the number of families for *A. baumannii* pangenome metrics without any impact in the interpretation.**

2-- Even though the principle of the PPanGGOLiN software is described in detail, the article does not provide much information about other existing software. It would be nice to have a few lines on the types of existing software (common features, novelties) and explain how PPanGGOLiN compares to them.

**Answer 1.2 : We agree with the reviewer #1 that the other existing methods could be more detailed.**

**Action 1.2 : We added sentences in the Background section of the manuscript to present in more detail three other statistical methods that can be used to partition pangenomes and their differences with PPanGGOLiN.**

3-- When estimating the persistent genome in comparison to the soft core approach (line 173 and onwards) you are introducing the idea that some gene families can belong to the persistent group even if they are observed in surprisingly low frequencies (line 234). Can you give some more concrete metrics on which percentage of the persistent genome would belong to the soft core and which to the shell?

**Answer 1.3 : The differences in the number of gene families in the persistent and the soft core genomes are illustrated in Figure 3 with the values available in supplementary file S11 (former name S10). In all studied species, the number of persistent gene families is greater than or equal to the soft core with an average of 11% (SD=9%) of additional families. Furthermore, persistent gene families include those of the soft core with the exception of very few gene families (only 12 gene families in total for all studied species which is negligible). For some species, such as Pseudomonas stutzeri, *Clostridium perfringens*, *Clostridium botulinum* and Colinsella sp., the size of the soft core genome is unexpectedly small and represents less than 55% of the genomes whereas it is above 75% for the PPanGGOLiN persistent. For the first three, it could be due to sampling effects and species heterogeneity. For the last one (Colinsella sp.), it could be explained because the species is made of incomplete genomes from metagenomes (i.e. MAGs) that were submitted as complete genomes in GenBank.**

**Action 1.3 : We added sentences at the beginning of the “Estimation of the persistent genome in comparison to the soft core approach” subsection of the manuscript. We also added a supplementary file S1 showing all the distributions of the frequencies of persistent, shell and cloud gene families in each species.**

4-- My main concern with the article is regarding the interpretation of the idea of partitions. In lines 306-307 for example, the large number of partitions is assumed to be due to non-homogeneous subclades. Could variation in partitions also be influenced by genome sampling? And taking this thought one step further, have you tried comparing the partitions that are generated in case of genus or phylum pangenomes? How about in the same example but by gradually adding more genomes in the analysis?

**Answer 1.4 : Indeed, genome sampling is one of the main limitations of pangenome studies. We showed, through rarefaction curves with IQR area and gamma-tendency values, that the estimation of the persistent genome by PPanGGOLiN seems to be resilient to sampling. For the shell component, the sampling effect can be important as the shell reflects by definition the diversity of the considered strains. PPanGGOLiN does not make any normalization of sampling and this could be an improvement of the method but we think that this issue should first be examined from a biological perspective. As suggested by Brockhurst et al. 2019, approaches to sample ecologically coherent microbial populations need to be considered. We tried PPanGGOLiN at the genus level and it produced reliable results but we did not perform a comprehensive analysis to discuss that point.**

**Action 1.4 : We added sentences in the Conclusion section about the current limitation of pangenome approaches in regards to sampling.**

5-- Some parts of the methods section do not have such a good use of the english language as the rest of the article. Below is a list of a few typos but it would be a good idea to scan the whole methods section for grammatical errors and typos.

line 287: due --> due to

line 546: share together --> shared

line 564: annotation --> annotations

line 565: there are --> they were

**Action 1.5 : We made the corrections asked by reviewer #1 and improved the english of the Methods section.**

**Reviewer #2:** This paper describes a method for using graph structures to model bacterial pan-genomes, in terms of gene presence and absence. This approach is different from conventional approaches -it is aesthetically pleasing, and kind of makes intuitive sense. However, I'm not sure that finding different results with a different method necessarily means that this is a 'better' method. Yes, it's different - but benchmarking is difficult. So I think that in a nutshell, the method is good, and should be published, but I do not think that the authors have convinced me that their method is necessarily 'better'.

**Answer 2.0 : We thank the reviewer #2 for his comments that are favorable to the publication of our article.**

**We agree that there is no method close enough to our approach to make a fair benchmark. However, the comparison with the soft core approach (that is widely used in the community) gives a good indication of the added-value of our results in the prediction of the persistent genome.**

**Action 2.0 : We suppressed the word ‘better’ in the authors summary, at line 245 (line 226 in the former version) and at line 408 (line 391 in the former version).**

The figures are good, but could be improved (especially the figure legends could be more clear) - Figure 1 is a nice overview, and the 'mobilome' in figure 2 is nicely visualized (although a black background is not optimal - white backgrounds can often give better visualizations). I like representing the species core-genomes on a single page, like Figure 3.

**Answer 2.1 : We thank the reviewer for the positive assessment of our figures. For figure 2, we tried a white background but the cloud nodes are more difficult to visualize. Thus, we prefer to keep the black background if there is no technical reserves from the journal editorial policy. We agree that captions could be improved.**

**Action 2.1 : We improved the captions of the figures.**

Lines 82-83 - the authors ASSUME that horizontally transferred genes ALWAYS go in to a few hot spots, and 'persistent genes' have conserved synteny (a word that is curiously missing from the entire manuscript!). Is it possible for a conserved gene to have different neighbors??

**Answer 2.2 : We agree that the term ASSUME may be too strong as of course conserved genes may have different neighbors, and that while most horizontally transferred genes tend to go into a few hotspots according to the literature, it is wrong to think that all of them do so. This is more a general observation than a strict PPanGGOLiN requirement. We also agree that the world synteny is missing.**

**Action 2.2 : We replace the term ASSUME by FAVOR at lines 88 (84 in the former version) and 517 (491 in the former version). We added the term ‘conserved synteny’ at line 86 (82 in the former version).**

Lines 151-152 - this seems contradictory - if their method proposed can be used for low quality genomes, then why is it necessary to throw out the low-quality genomes?

**Answer 2.3 : We understand the apparent contradiction raised by reviewer #2. The main goal of the QC step is to filter out genomes that have an apparently wrong species definition due to taxonomic assignation errors or to inaccurate species delineation in the NCBI taxonomy. Furthermore, we removed potentially contaminated genomes according to the QC of GenBank. Taxonomic errors and contamination can have a major impact on the analysis of pangenomes. The last filter regarding assembly metrics is here to remove aberrant assemblies that may indicate technical issues during the sequencing/assembly process.**

**Action 2.3 : We added some precisions in the manuscrit on the reasons underlying the QC in Results (line 160, 151 in the former version) and Methods sections (line 567, 542 in the former version).**

Lines 190-191 - Yes, the 'most cited software' assumes 95% - but this does not necessarily make it correct or the 'best'. There are mathematical methods for testing this - see for example the "kneedle" method. Satopaa, V., Albrecht, J., Irwin, D., and Raghavan, B. (2011).

Finding a “kneedle” in a haystack: Detecting knee points in system behavior. In 2011 31st International Conference on Distributed Computing Systems Workshops, pages 166–171.

**Answer 2.4 : We totally agree with reviewer #2 that the threshold of 95% is not necessarily the ‘best’ one and that the word ‘admitted’ is confusing.**

**Action 2.4 : We reformulate the sentence in the manuscript about the use of this threshold. (line 186, 191 in the former version).**

**Other changes :**

- **We added Fig. S1 showing the density distributions of the frequency of gene families from each partition within the 88 most abundant species in GenBank. Consequently the number of all the supplementary figure is shifted.**
- **We improved Fig. S8 (former name : Fig. S7) as we noticed a few mistakes in the legend (did not impact interpretation)**
- **We improved the legend of the Fig. 7.**
- **We corrected formulas indicating the number of parameters in the ‘Selection of the optimal number of partitions’ section. They were inverted in the manuscript but were properly written in the source code thus it had no impact on the results.**
- **We improved the english throughout the manuscript**
